# The Prevalence of *Trichinella spiralis* in Domestic Pigs in China: A Systematic Review and Meta-Analysis

**DOI:** 10.3390/ani12243553

**Published:** 2022-12-15

**Authors:** Huifang Bai, Bin Tang, Weidong Qiao, Xiaoxia Wu, Mingyuan Liu, Xuelin Wang

**Affiliations:** State Key Laboratory for Zoonotic Diseases, Key Laboratory for Zoonosis Research, Ministry of Education, College of Veterinary Medicine, Jilin University, Changchun 130062, China

**Keywords:** foodborne disease, parasitic disease, trichinellosis, zoonosis

## Abstract

**Simple Summary:**

As a pathogen of trichinellosis, *Trichinella spiralis* is a foodborne zoonotic nematode that can infect more than 100 species including mammals, birds, and reptiles. Pigs infected with *T. spiralis* are the primary host for disseminating it to humans. Therefore, a meta-analysis was performed here to assess the prevalence of *T. spiralis* in domestic pigs in China. After considering 43 different studies, including a total sample size of 551,097 pigs, these results indicated that *T. spiralis* were still prevalent in some areas in China and the highest prevalence region was Guangxi.

**Abstract:**

The meta-analysis was performed to assess the prevalence of *T. spiralis* in domestic pigs in China. The potential studies from seven databases (Pubmed, Web of science, Scopus, Google Scholar, CNKI, Wanfang, CBM) were searched. I^2^, Cochran’s Q statistic and the funnel plot and Egger’s test were used to assess heterogeneity and publication bias, respectively. In this study, a total of 179 articles were captured in the initially screened. Of these, we finally obtained 39 significant articles (including 43 studies involving in 551,097 pigs) for the final analysis. We calculated using a random-effects model, and we found the overall infection rate was 0.04 (95% CI 0.03–0.06). The highest prevalence region was Guangxi. The funnel plot and Egger’s test showed no publication bias in our meta-analysis. In addition, this high heterogeneity index was suggestive of potential variations which could be due to regions, quality scores, detection methods, publication years, or samplings. These results indicated that *T. spiralis* were still prevalent in some areas in China. This highlights the need for an increased focus on implementing affordable, appropriate control programs to reduce economic losses and *T. spiralis* infection in domestic pigs in China.

## 1. Introduction

As a pathogen of trichinellosis, *Trichinella spiralis* (Owen, 1835) is a foodborne zoonotic nematode that can infect more than 100 species including mammals, birds and reptiles [1,2]. *T. spiralis* infection is fatal in humans infected with a large number of larvae [3,4]. The nematode of pork products can be thermally inactivated at 76.7 °C, a cooking internal temperature recommended by US Food and Drug Administration (USDA) [5]. Humans are infected by eating raw or undercooked pig meat and sausage containing the larvae of *T. spiralis*. The nematode has caused serious physical and financial burdens on public health [6]. *T. spiralis* infection have two phases in the human, the enteric or gastrointestinal phase and the systemic (parenteral) phase. Gastrointestinal symptoms are the first symptoms of trichinellosis. Symptoms include abdominal pain, diarrhea, nausea, and vomiting. Muscle pain is a common complaint, chiefly in the mid-abdomen, face (masseter), and chest (intercostal muscles). During the second phase, larvae enter the lymphatic circulation and then into the blood, reaching skeletal muscles, myocardium, and the brain which are high in oxygen content. This phase leads to systemic symptoms like fevers, myositis, myalgias, periorbital edema, and can even cause myocarditis and encephalitis [7]. According to the estimation of World Health Organization (WHO) based on data from 2010 to 2015, parasitic diseases resulted in 48.4 million cases and 59,724 deaths annually, resulting in 8.78 million disability-adjusted life years (DALYs), and it is further estimated that 48% of these parasitic diseases were foodborne [8]. According to Murrell and Pozio [9], from 1986 through 2009, there were 65,818 trichinellosis cases and 42 deaths reported from 41 countries. As the definitive hosts, pigs play an important role in the whole life cycle of *T. spiralis* [10]. According to preliminary statistics, the production and consumption of pig meat in China is the highest in the world [11]. As a result, it is estimated that approximately 400,000 people are at the risk of infection with *T. spiralis* in China [12,13].

The traditional current line of trichinellosis treatment commonly employs drugs belonging to benzimidazole derivatives [14]. Albendazole (ABZ) is considered the drug of choice in the treatment of trichinellosis [15]. However, in addition to the emerging resistance, ABZ have low water solubility resulting in reduced bioavailability [16]. Although the therapy methods of trichinellosis have been improved, the controlling key of this disease is still decreasing its definitive-host infection rate [17]. *T. spiralis* is slightly virulent to pigs and hardly causes obvious symptoms. Normally, the larvae parasitized in the striated muscles and intestines of pigs [18]. However, in the early stage of large dose infection, infected pigs may show symptoms such as loss of appetite, diarrhea, vomiting, and so on. In the middle and late stage, infected pigs may show symptoms such as pain or paralysis, temperature rise, emaciation, chewing and swallowing disorders, hoarseness, and so on [7,19].

Currently, as a result of drug-resistance escalation and immune failure [20,21,22,23], pig industries have to alter approaches to controlling the parasitic nematodes by developing new vaccines or drugs against *T. spiralis* infection in pigs. The lack of information on the epidemiology also lead to difficulty in the development of new vaccines, drugs, and the diagnosis technology of trichinellosis. Trichinellosis is not only fatal; there are severe debilitating morbidity associated with the detrimental effects of drug resistance which can lead to prolonged drug treatment, negative socioeconomic effects, and adversely impact normal daily productive activities [24,25]. As a result, based on numerous impacts of trichinellosis on animal welfare, the economy, and public health, further considerations and research are deemed to be a desideratum for epidemiological approaches and monitoring programs in China.

Until this study, no attempt has been made to integrate all published studies and reports to derive a robust prevalence estimate of *T. spiralis*. As a result of the estimation, the aim of this study was to estimate the prevalence and distribution of *T. spiralis* in domestic pigs in China. This review will also help to evaluate the suitable sample size of vaccine and drug experiments in the high prevalence regions and validate diagnostic tests and vaccine developments for the prevention and control of trichinellosis.

## 2. Methods

The protocol of this systematic review was defined in advance and registered with PROSPERO (International Prospective Register of Systematic Reviews) (ID: CRD42021270969). The systematic review and meta-analysis in the study was conducted according to the guidelines provided by PRISMA 2020 (Preferred Reporting Items for Systematic Reviews and Meta-analysis 2020) [26]. The PRISMA 2020 checklist (Text S1) was followed to ensure the inclusion of relevant information and maintain study standards.

### 2.1. Literature Search

To evaluate the prevalence of *T. spiralis* in domestic pigs in China, the potential studies from 7 databases (Pubmed, Web of science, Scopus, Google Scholar, CNKI, Wanfang, CBM) were searched. The modified searches were performed by various combinations of the following terms using Boolean operator “AND” and “OR”: (*Trichinella spiralis* OR *Trichinella* spp. OR *Trichinella* OR Trichinellosis) AND (prevalence OR distribution OR epidemic OR incidence OR frequency OR occurrence OR detection OR identification OR characterization OR investigation OR survey OR rate) AND (China OR Chinese OR Asia OR Asian). The reference lists of selected articles were also screened manually and appropriate articles were included. Full-text articles were downloaded or obtained through library resources. No attempt was made to identify unpublished reports.

### 2.2. Selection of Studies

All selected articles had to meet the following inclusion criteria: (i) cross-sectional, cohort, or case-control studies; (ii) the studies were published between January 1990 and December 2021; (iii) the language was limited to Chinese or English; (iv) full-text articles were published; (v) infection cases were from China; (vi) reported as animal level prevalence data, not laboratory infected animals; (vii) exact total numbers and positive cases numbers were given; (viii) animal numbers higher than 200; (ix) the animals must be domestic pigs or swine. Studies were excluded if they did not fulfill all of these criteria. Furthermore, if the same study data were published in both English and Chinese sources, the articles with less detailed information would be excluded from our study. When any authors found articles difficult to judge, the corresponding author was consulted, and differences were discussed until a consensus decision on whether to include or exclude the article was reached.

### 2.3. Quality of the Studies

The quality of the selected publications was accessed according to the criteria derived from the Grading of Recommendations Assessment, Development and Evaluation (GRADE) method [27]. The quality of the publications was graded using a scoring approach (Text S2). This action was performed by three independent authors. Any difference in opinion among authors or uncertainty was discussed with the corresponding author and all authors had to extract data according to the result of the discussion. A checklist including 8 items was considered for thorough reporting of observational studies. These items were related to the article’s title, abstract, introduction, materials and methods, results, and discussion sections. The score under 2 (≤2) was considered a low quality, between 2 and 5 (>2, ≤5) were middle, and >5 was high [28].

### 2.4. Data Analysis

The statistical software used in the analysis was R software version 3.6.3 (New Zealand, University of Auckland, Auckland, New Zealand). Before preforming the meta-analysis, we used four methods to convert the observed proportions: The logarithmic conversion (PLN), the logit transformation “PLOGIT”, arcsine transformation (PAS), and Freeman–Tukey double arcsine transformation (PFT). We performed a normal distribution test on the observed proportions and the transformation proportions. We first assumed that the overall data obeyed a normal distribution. The maximum value of the statistic W is 1, and the closer the value of W is to 1 indicates that the sample matches the normal distribution. If *p* < 0.05, the null hypothesis is rejected, and the dataset does not conform to the normal distribution. When W is close to 1 and *p* > 0.05, the null hypothesis cannot be rejected, and the dataset matches the normal distribution. After transforming the observed proportions, all analyses were conducted using the transformed proportion as the effect size statistic and the inverse of the variance of the transformed proportion as the study weight [29]. According to the above, in this analysis, estimated pooled prevalence and 95% confidence intervals (CI) were calculated with PFT. Heterogeneity testing was performed using the I^2^ and Cochran’s Q statistic methods (represented as χ^2^ and *p* value) [30,31]. A significant value (*p* < 0.05) in the analysis suggested a real effect difference. The I^2^ values of 25%, 50%, and 75% were considered as low, moderate, and high heterogeneity, respectively. The risk of study publication bias was assessed using the funnel plots, and the Egger’s regression test. We also used trim and fill analysis and sensitivity analysis to assess the stability of our study [32].

Furthermore, a significant value (*p* < 0.05) in the analysis suggested a real effect difference. The potential sources of heterogeneity (I^2^ > 50%) were further investigated by subgroup analysis and meta-regression analysis. Five potential sources of heterogeneity were examined: regions, detection methods, samplings, publication years, and quality scores. The Q and I^2^ statistics values were calculated for each subgroup to determine the effective factors on the prevalence *T. spiralis* and heterogeneity about all included studies [33].

## 3. Results

### 3.1. Search Results and Eligible Studies

**Figure 1:**
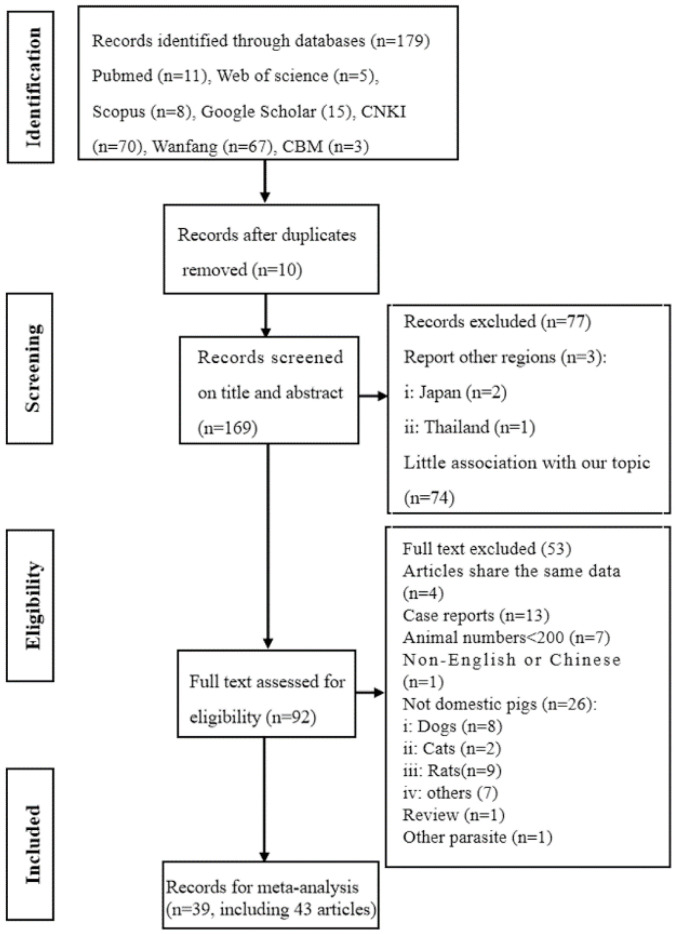
The flow diagram of search and selection of relevant articles for a systematic review on the prevalence of *Trichinella spiralis* in domestic pigs in China.

A flow diagram depicted the study selection process in the flow chart (Figure 1). In this study, totally 179 articles were searched after retrieval from 7 databases, and 169 papers were identified after the removal of duplicates. After screening on title and abstract, 77 articles were further excluded, 2 papers from Japan, 1 paper from Thailand, and 74 articles have little association with our topic. Of these, 53 articles were further excluded due to the following reasons: 4 articles shared the same data, 13 articles were case reports, the animal numbers were less than 200 in 7 articles, 1 article was non-English and Chinese, and 26 articles, of which 8 were dogs, 2 were cats, 9 were rats, 7 were other animals, did not refer domestic pigs, 1 article was a review, and 1 article concerned aother parasite. Finally, a total of 39 articles, including 43 studies were used for meta-analysis. The complete list of included articles can be found in Table 1. Each study used a cross-sectional design. There were 6 studies from Qinghai, 1 from Gansu, 9 from Henan, 2 from Hubei, 10 from Guangxi, 1 from Guizhou, 1 from Tibet, 4 from Yunnan, 1 from Sichuan, 1 from Heilongjiang, 2 from Inner Mongolia, 1 from Shanxi, 1 from Beijing, 1 from Hebei, 1 from Jiangsu, and 1 from Shandong, respectively.

### 3.2. Pooling and Heterogeneity Analysis

**Figure 2:**
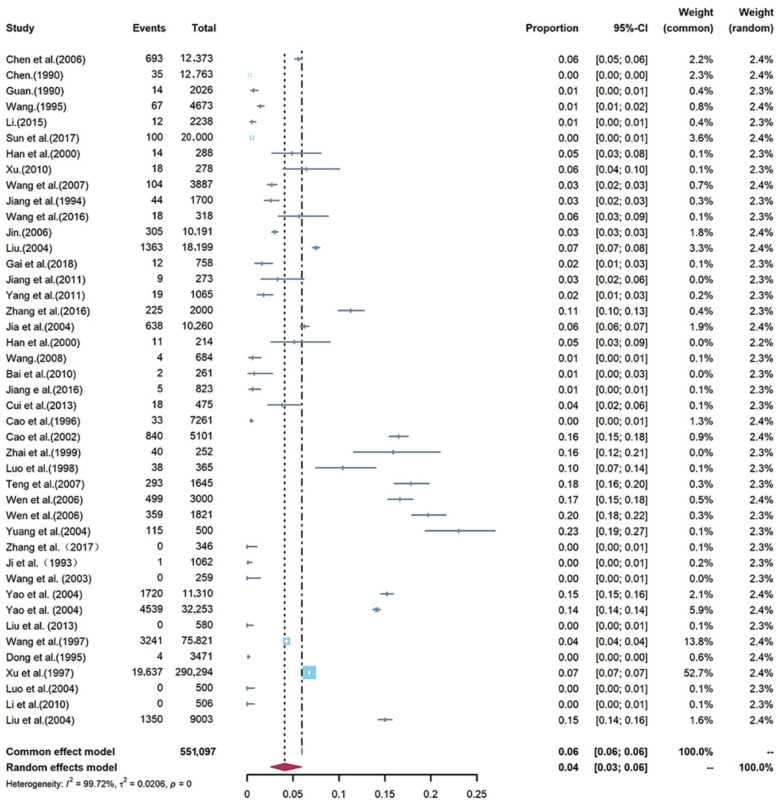
Forest plot showing the pooled prevalence of *Trichinella spiralis* in domestic pigs in China. The length of the horizontal line represents the 95% confidence interval and the diamond represents the summarized effect [34,35,36,37,38,39,40,41,42,43,44,45,46,47,48,49,50,51,52,53,54,55,56,57,58,59,60,61,62,63,64,65,66,67,68,69,70,71,72].

The pooled prevalence estimates of *T. spiralis* infection in domestic pigs with individual studies were showed in a forest plot (Figure 2). A substantial heterogeneity was observed among studies (*p* < 0.05; I^2^ = 99.72%). The overall infection rate calculated using a random-effects model was 0.04 (95% CI 0.03–0.06; 36,439/551,097) and lower than 1.97% as reported by Wang et al. [73].

**Figure 3:**
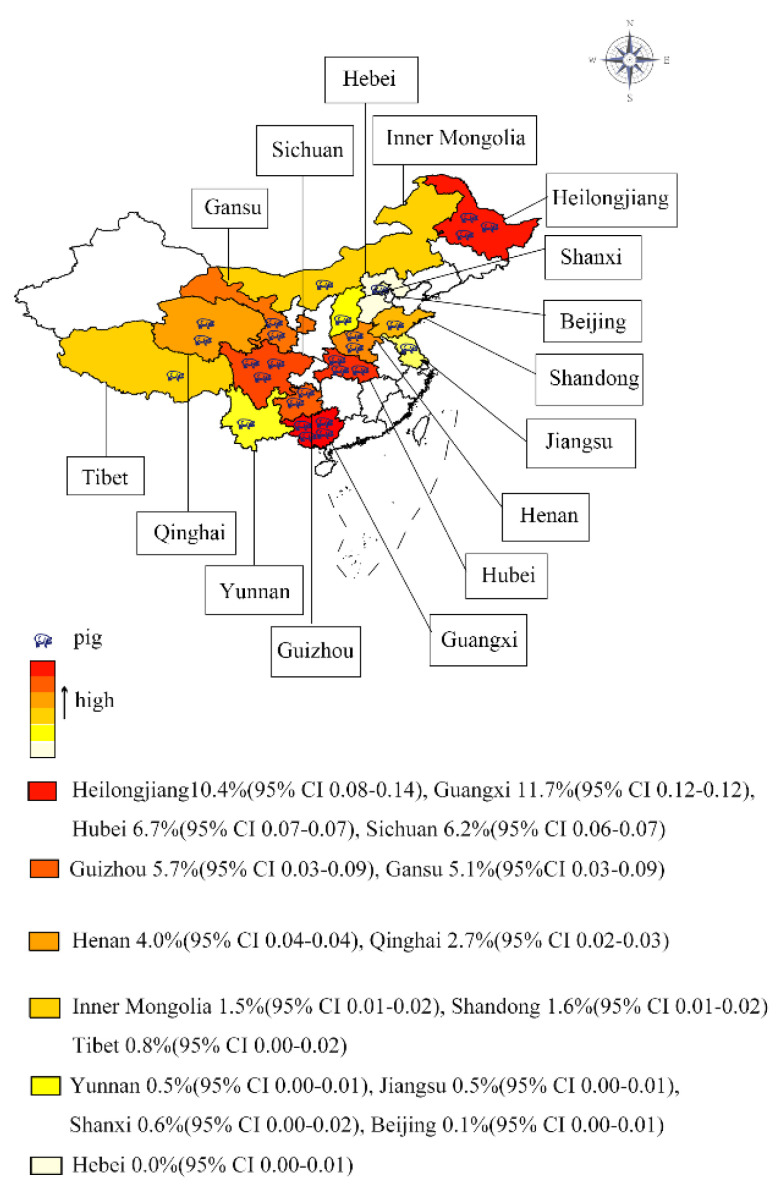
Distribution map of *Trichinella spiralis* in domestic pigs in China.

The estimates of infection rates for different subgroups and heterogeneity were presented in Table 2 and Figures S1–S5. All pooled infection rates for each subgroup were calculated using a random-effects model because of the observed high heterogeneity of subgroups among the studies. Infection rates varied across different geographical regions in China. In the region subgroups, the highest point estimate was in Central South (0.06, 95% CI 0.03–0.09; 35,072/484,584), especially in Guangxi (0.12, 95% CI 0.12–0.12; 111,335/97,196) (Figure 3). At the region level, there was no prevalence in Hebei as we described. Moreover, we further analyzed the studies by years. The different publication years showed a significantly different (*p* < 0.05) infection rate: the prevalence in 2000 to 2008 was the highest (0.08, 95% CI 0.05–0.13; 12,822/120,986), followed by before 2000 (0.02, 95% CI 0.01–0.04, 23,179/400,190), and the lowest was 2008 and later (0.02, 95% CI 0.01–0.03; 438/29,921). Based on study detection methods, P&E (parasitology and enzyme-linked immunosorbent assay) showed the highest detection rate (0.08, 95% CI 0.04–0.13; 12,087/128,475). We also conducted other subgroup analyses such as sampling. The result showed the B&S (biopsy and serology) sampling was highest (0.08, 95% CI 0.05–0.13; 12,124/127,689). Finally, in terms of quality levels, the estimate was highest in the middle score (0.05, 95% CI 0.02–0.08; 30,938/452,599). The univariate meta-regression showed that regions, publication years, samplings, detection methods, and quality scores may be major sources of heterogeneity (*p* < 0.05).

### 3.3. Publication Bias and Sensitivity Analysis

**Figure 4:**
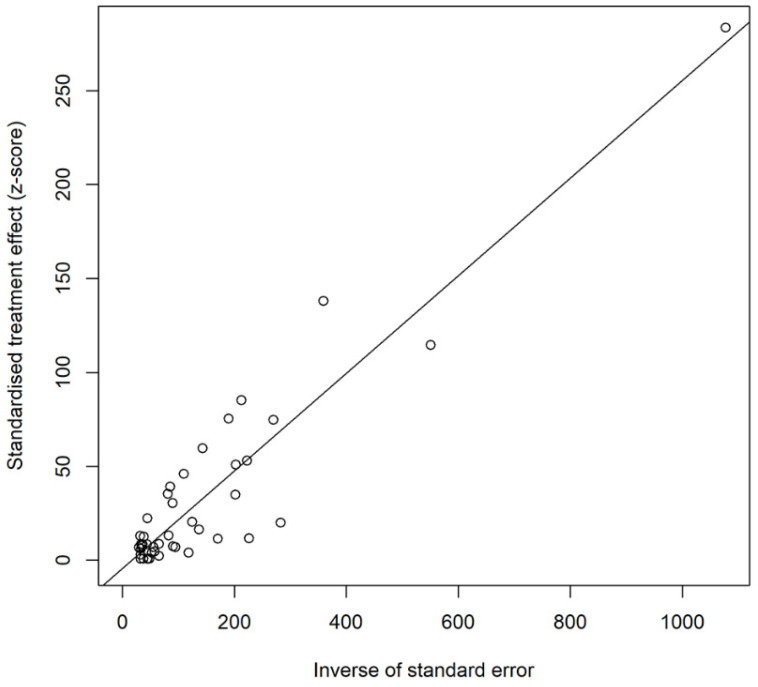
Egger’s test for publication bias in *Trichinella spiralis* infection in domestic pigs in China.

**Figure 5:**
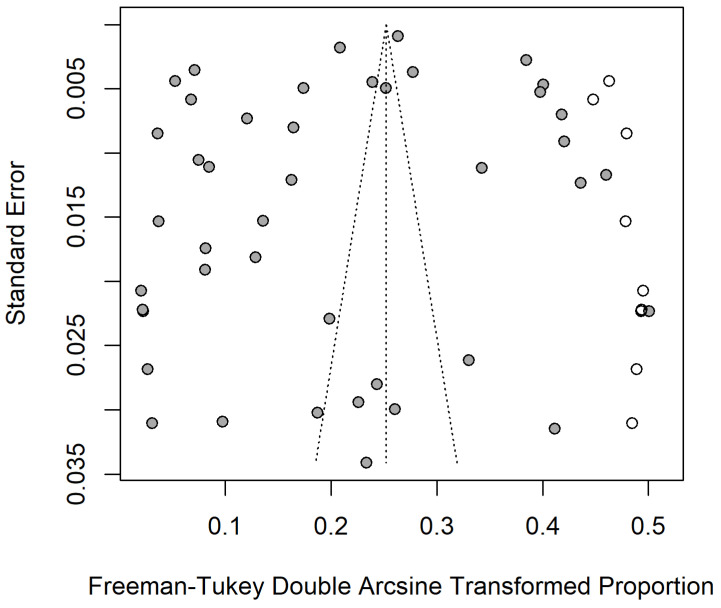
Funnel plot with trim and fill analysis of the publication bias in *Trichinella spiralis* infection in domestic pigs in China.

We used PFT to convert the raw rate to ensure the data were closer to a normal distribution (Table 3). As the funnel plot showed, the studies that we included might have publication bias or small-sample size bias (or small-study effects bias) (Figure S6). The result of Egger’s test revealed that there was no publication bias (*p* = 0.2374 > 0.05) (Figure 4). Therefore, the studies we included may not have publication bias, but a small sample size bias cannot be ruled out [74,75]. The result of the trim and fill test showed that there were nine studies which were added (the point estimate was 0%) and the pooled estimate was finally changed (Figure 5). The sensitivity analysis indicated that the pooled prevalence was not significantly affected by each study after omitting any one study at a time, so we believed that the stability of the results was reliable and rational (Figure S7).

## 4. Discussion

Trichinellosis is a seriously neglected foodborne zoonotic disease with a worldwide prevalence [76]. An overview of knowledge on the geographical distribution and burden of *T. spiralis* will offer a better understanding of its impacts on animal production and risk to public health [77,78,79]. We conducted a meta-analysis to estimate the prevalence of *T. spiralis* in domestic pigs in China and assess the potential factors. In this study, the overall infection rate was 0.04 (95% CI 0.03–0.06) but the highest prevalence region was 11.7% in Guangxi, which was higher than the prevalence region of China reported by Cui et al. [80]. The result was consistent with previous studies. Studies conducted in neighboring countries found the seroprevalence to be 2.5% in Rural Cambodia, 5.6% in Vietnam, and 2.1% and 14.4% in different provinces in Lao PDR [81,82,83,84]. The infection rate may vary significantly within and between countries. The pig international trade represents one of the largest livestock markets in the world [85]. The risk of trichinellosis linked to pig consumption is higher in China than in neighboring countries [86,87,88].

There was high heterogeneity in prevalence levels in domestic pigs across China mainland among the eligible studies, but no significant publication bias was found at cut off level of 0.05 by Egger’s test or trim and fill analysis. This high heterogeneity index was suggestive of potential variations, which could be influenced by regions, quality scores, detection methods, publication years, or samplings. To trace the source of heterogeneity, articles were first divided into six subgroups. There was a significantly higher prevalence in Central China (*p* < 0.05), although further meta regression analysis of the region subgroups did show no significant differences (*p* > 0.05). The epidemiology of *trichinellosis* are the results of many geographical, ecological, and social interactions which may explain some of these differences. The majority of outbreaks attributed to domestic pigs have been traced to pigs raised in small farms or backyards, often outdoors, where poor husbandry conditions place pigs at high risk [85]. However, the growing popularity of free-range pig production, because it involves varying degrees of outdoor exposure and even direct contacting with reservoir hosts such as foxes, raccoon dogs, or wild boars/feral pigs, has raised concerns that pastured pigs may have an increased risk of spillover of *T. spiralis.* Correspondingly, in Central China, pigs industry was especially widespread and more backyard or outdoor free-ranging pigs are maintained than in other regions of the country. In China, pig *T. spiralis* infection is still principally transmitted by garbage (i.e., feeding pigs with swills containing raw pork scraps). *T. spiralis*-infected pigs predominantly also came from small backyard farms where animals were raised under poor hygienic conditions and outdoor free-ranging pigs that were fed raw waste products or animal carcasses [89]. Prevention and controlling infection with *T. spiralis* should be seriously considered in these regions, and the traditional pig-rearing mode should be improved.

Most of studies in our analysis (n = 35) were of high and middle quality; therefore, this study can reflect the basic prevalence of *T. spiralis* among domestic pigs in China. The reason for losing points in some studies was a failure to distinguish the region. The results showed the difference of prevalence rates was significant between studies of different quality, and we found the estimate was highest in middle score (>2 to ≤5). In addition, results of the univariate regression analysis suggested that the quality of articles may be a source of heterogeneity in this study. The result was consistent with the report by Gong [90].

In the study, the infection rate of *T. spiralis* was identified by different methods with significant difference in the reported prevalence (*p* < 0.05). In 2016, the World Organization for Animal Health (OIE) reported that the digestion method is the best testing method for diagnosing trichinellosis [91]; however, detection rate was lowest in this method in our analysis. Although this method was simple and inexpensive, it was not sensitive; it was easy to confused *T. spiralis* with other microorganisms and increase the false positive rate. Moreover, compared to microscopic examination, the digestion method is the reliable method, but it is laborious, biohazardous, and could raise ethical issues [92,93]. We also found that the most common method (ELISA) was still lower; however, this method, without optimizing antibody concentration, is fast, reliable, sensitive, and suitable for large-scale testing. The test result of the method will largely depend on operation [94]. Additionally, the manufacturers, and cross-reaction between species of *T. spiralis* or other helminth antigens, can also lead to false positives [95]. The P&E methods were highest, which may improve the detection rate of *T. spiralis*. The classification of sampling types confirmed the infection rate used by B&S. As Eslahi et al. [96] stated, serology has been shown as a better and more efficient detection tool than biopsy. Serological techniques were the most frequently used methods for trichinellosis diagnosis. However, higher rates of infection were detected by the combination with biopsy [97]. Serology diagnostic tests was the most appropriate diagnostic method with a combination of serology, molecule, and biopsy approaches [11,74].

The study demonstrated that the estimated pooled prevalence of *T. spiralis* in domestic pigs in China between 2000 to 2008 was the highest. The infection rate was decreasing after 2008. The microscopy techniques used by different authors before 2000, the more sensitive methods such as LAMP, PCR-based, and ELISA detection methods were used in the 2000s. As a result, there was greater *T. spiralis* prevalence surveyed by government programs in China after 2000. The reasons led to the higher infection rate which is consistent with previous hypotheses. *T. spiralis* ranked first in the Food and Agriculture Organization of the United Nations (FAO) and WHO international trade list of 24 parasites according to nine global criteria in 2012 [98]. With the implementation of National Mid- and Long-Term Animal Disease Control Plan (2012–2020) and Biosecurity Law (2021) in China, the infection rate was decreasing and there was a positive contribution to changing dietary habits and environments and public awareness after 2008 [74].

Understanding the distribution and associated risk factors of *T. spiralis* was essential to improving public health. The potentially increasing risk of pig trichinellosis may influence the re-emergency occurrences of human infection in China. It should encourage government administration to implement more measures to control trichinellosis of domestic pigs. Overall, these results showed that special attention should be paid to public hygiene and animal care in order to prevent *T. spiralis* infection in China.

## 5. Limitations

There are some limitations to this study. First, the number of eligible studies was small for available analysis on *T. spiralis* prevalence. Some studies had a small sample size which may have affected the validity of the overall estimates. We also consider that more than 700 million pigs are produced annually in China. In this meta-analysis, the data we obtained was limited. In order to improve this search, we will continue to pay more attention to new reports on China in the future. Second, although the publication bias was not detected, the unreported articles might lead to an uneven coverage and confounding factors affecting differences in *T. spiralis* infection prevalence among different regions by the study. This highlights the need for improving disease surveillance and clearly identifying trichinellosis’s geographic heterogeneities by epidemiology nationwide. Third, overall heterogeneity for all pooled prevalence estimations was high, and should be interpreted with caution. Furthermore, heterogeneity remained high after stratification by regions, quality scores, detection methods, publication years, or samplings, suggesting that there were significant residual effects of unmeasured variables. Fourth, no more risk factors were analyzed. Further studies are required to exclude other influencing factors, such as rearing methods, farming scale, and sampling seasons, which might have been sources of high heterogeneity.

## Figures and Tables

**Table 1:** Baseline characteristics of included studies of *Trichinella spiralis* infection in domestic pigs in China.

ID	Study	Period	Location	P^e^ (%)	Sampling			Detection Methods			Score
				N	Total	B	S	P	M	E	
1	[34]	1993~2004	Hubei	693 (5.60)	12,373	Y		Y			7
2	[35]	1998	Yunnan	35 (0.27)	12,763	Y		Y			5
3	[36]	1995.3~1995.7	Qinghai	14 (0.69)	2026	Y		Y			4
4	[37]	NA	Guangxi	67 (1.43)	4673		Y	Y		Y	2
5	[38]	2013.9~2014.12	Henan	12 (0.54)	2238	Y		Y	Y		7
6	[39]	NA	Inner Mongolia	100 (0.50)	20,000	Y	Y	Y		Y	7
7	[40]	NA	Qinghai	14 (4.86)	288		Y			Y	2
8	[41]	NA	Henan	18 (6.47)	278	Y		Y	Y		7
9	[42]	2003.1~2005.12	Henan	104 (2.93)	3887	Y	Y	Y	Y		7
10	[43]	1965	Yunnan	44 (2.58)	1700	Y		Y			5
11	[44]	2014.9~2015.8	Guizhou	18 (5.66)	318	Y				Y	5
12	[45]	2002.6~2002.9	Guangxi	305 (2.99)	10,191	Y	Y	Y		Y	6
13	[46]	2001.7~2003.11	Guangxi	1363 (7.49)	18,199	Y	Y	Y		Y	7
14	[47]	2015~2017	Shandong	12 (1.58)	758	Y	Y	Y		Y	7
15	[48]	2010~2011	Henan	9 (3.30)	273	Y		Y			8
16	[49]	2010.12	Qinghai	19 (1.78)	1065		Y			Y	4
17	[50]	2015.1~2015.7	Inner Mongolia	225 (11.25)	2000		Y			Y	7
18	[51]	NA	Sichuan	638 (6.22)	10,260	Y	Y	Y		Y	4
19	[40]	NA	Gansu	11 (5.14)	214		Y			Y	2
20	[52]	NA	Shanxi	4 (0.58)	684	Y		Y			2
21	[53]	2009.8~2010.1	Tibet	2 (0.77)	261	Y	Y	Y		Y	6
22	[54]	2014–2015	Henan	5 (0.61)	823	Y		Y			5
23	[55]	2010.2~2011.2	Henan	18 (3.79)	475	Y		Y			6
24	[56]	1993.11	Jiangsu	33 (0.003)	7261	NA		NA			3
25	[57]	2001.7~2002.6	Guangxi	840 (16.47)	5101	Y	Y	Y		Y	4
26	[58]	1997.5~1997.7	Qinghai	40 (15.87)	252	Y		Y			3
27	[59]	NA	Heilongjiang	38 (10.41)	365		Y			Y	2
28	[60]	2004.1~2005.12	Guangxi	293 (17.81)	1645	Y	Y	Y		Y	7
29	[61]	2004.1~2004.12	Guangxi	499 (16.63)	3000	Y	Y	Y		Y	6
30	[61]	2005.1~2005.10	Guangxi	359 (19.7)	1821	Y	Y	Y		Y	6
31	[62]	2004.1~2004.3	Qinghai	115 (23)	500	Y		Y			3
32	[63]	NA	Henan	0 (0)	346	Y		Y			3
33	[64]	NA	Beijing	1 (0.09)	1062		Y			Y	2
34	[65]	2002–2003	Hebei	0 (0)	259	Y		Y			6
35	[66]	2001	Guangxi	1720 (115.21)	11,310	Y	Y	Y		Y	5
36	[66]	2002	Guangxi	4539 (14.07)	32,253	Y	Y	Y		Y	5
37	[67]	2010.5–2011.5	Henan	0 (0)	580		Y			Y	6
38	[68]	1995	Henan	3241 (4.27)	75,821	Y		Y			4
39	[69]	1990.5–1990.7	Qinghai	4 (0.12)	3471	Y		Y			2
40	[70]	1985–1993	Hubei	19,637 (6.76)	290,294	Y		Y			3
41	[71]	NA	Yunnan	0 (0)	500	Y		Y			2
42	[72]	2003.8	Yunnan	0 (0)	506	Y		Y			3
43	[46]	2001.7–2003.11	Guangxi	1350 (15)	9003	Y	Y	Y		Y	7

NA: data not applicable; P: parasitology; E: enzyme-linked immunosorbent assay; M: molecular; B: biopsy; S: serology; P^e^: positive rate, N/Total (%); Y: yes.

**Table 2:** Pooled prevalence of risk factors in *Trichinella spiralis* infection in domestic pigs in China.

Variable	No. of Studies	No. of Test	No. of Positive	Prevalence (%) 95%CI	Heterogeneity (Q)			Univariate	
								Meta-Regression	
					Q	I^2^ (%)	*p*	Coefficient (95% CI)	*p*
Region								0.501 (0.242,0.760)	<0.05
Northwest				0.03 (0.01, 0.10)	360.69	98.34	<0.01	−0.314 (−0.585, −0.043)	<0.05
Qinghai	6	7602	206						
Gansu	1	214	11						
Central South				0.06 (0.03, 0.09)	7119.89	99.72	<0.01	−0.256 (−0.521, 0.009)	>0.05
Henan	9	84,721	3407						
Hubei	2	302,667	20,330						
Guangxi	10	97,196	11,335						
Southwest				0.01 (0.00, 0.04)	1007.10	99.40	<0.01	−0.404 (−0.681, −0.128)	<0.05
Guizhou	1	318	18						
Tibet	1	261	2						
Yunnan	4	15,469	79						
Sichuan	1	10,260	638						
Northeast				NA	NA	NA	NA	NA	NA
Heilongjiang	1	365	38						
North China				0.01 (0.00, 0.05)	561.51	99.29	<0.01	−0.425 (−0.741, −0.109)	<0.01
Inner Mongolia	2	22,000	325						
Shanxi	1	684	4						
Beijing	1	1062	1						
Hebei	1	259	0						
East China				NA	NA	NA	NA	−0.269 (−0.586, 0.048)	>0.05
Jiangsu	1	7261	33						
Shandong	1	758	12						
Score								0.209 (0.070, 0.348)	<0.05
≤2 (low)	8	11,257	139	0.02 (0.00, 0.04)	211.72	96.69	<0.01		
>2 to ≤5 (middle)	17	452,599	30,938	0.05 (0.02, 0.08)	8287.33	99.81	<0.01		
>5 to ≤8 (high)	18	87,241	5362	0.05 (0.02, 0.08)	4993.45	99.66	<0.01		
Detection methods								0.176 (0.073, 0.278)	<0.01
P	17	403,066	23,859	0.02 (0.01, 0.05)	4255.20	99.62	<0.01		
E	8	5892	326	0.04 (0.01, 0.08)	421.94	98.34	<0.01		
P&E	14	128,475	12,087	0.08 (0.04, 0.13)	8124.97	99.84	<0.01		
P&M	3	6403	134	0.03 (0.00, 0.07)	64.02	96.88	<0.01		
NA	1	7261	33	NA	NA	NA	NA		
Sampling								0.195 (0.131, 0.259)	<0.01
B	20	405,900	23,907	0.02 (0.01, 0.04)	4504.68	99.58	<0.01		
S	8	10,247	375	0.03 (0.01, 0.07)	478.20	98.54	<0.01		
B&S	14	127,689	12,124	0.08 (0.05, 0.13)	7834.34	99.83	<0.01		
NA	1	7261	33	NA	NA	NA	NA		
publication year								0.281 (0.217, 0.345)	<0.05
≤2000	13	400,190	23,179	0.02 (0.01, 0.04)	1612.74	99.26	<0.01		
>2000 to ≤2008	16	120,986	12,822	0.08 (0.05, 0.13)	3974.11	99.62	<0.01		
>2008	14	29,921	438	0.02 (0.01, 0.03)	685.18	98.10	<0.01		
Overall		551,097	36,439						

CI: Confidence interval; NA: data not applicable; P: parasitology; E: enzyme-linked immunosorbent assay; M: molecular; P&E: parasitology and enzyme-linked immunosorbent assay; P&M: parasitology and molecular; B: biopsy; S: serology; B&S: biopsy and serology; *p* < 0.05 is statistically significant.

**Table 3:** Normal distribution test for the normal rate and the different conversion of the normal rate in *Trichinella spiralis* infection in domestic pigs in China.

Conversion Form	W	*p*
PRAW	0.82574	1.352 × 10^−5^
PLN	NaN	NA
PLOGIT	NaN	NA
PAS	0.935	0.01732
PFT	0.93684	0.02009

“PRAW”: original rate; “PLN”: logarithmic conversion; “PLOGIT”: logit transformation; “PAS”: arcsine transformation; “PFT”: double-arcsine transformation; “NaN”: meaningless number. “NA”: missing data; W; wilcoxon value; *p*: *p* value.

## Data Availability

All datasets are included in the manuscript or as supplementary files.

## Supplementary Materials

The following supporting information can be downloaded at: https://www.mdpi.com/article/10.3390/ani12243553/s1, Text S1: PRISMA checklist 2020; Text S2: Quality assessment checklist; Figure S1: Forest plot diagram showing *Trichinella spiralis* infection in domestic pigs in China in different regions; Figure S2: Forest plot diagram showing *Trichinella spiralis* infection in domestic pigs in China in different publication year; Figure S3: Forest plot diagram showing *Trichinella spiralis* infection in domestic pigs in China in different detection methods; Figure S4: Forest plot diagram showing *Trichinella spiralis* infection in domestic pigs in China in different scores; Figure S5: Forest plot diagram showing *Trichinella spiralis* infection in domestic pigs in China in different samplings; Figure S6: Funnel plot displaying prevalence data for all included articles of *Trichinella spiralis* infection in domestic pigs in China; Figure S7: Sensitivity analysis of *Trichinella spiralis* infection in domestic pigs in China. Reference [99] is cited in the supplementary materials.
