# Supplementary material for: The Prevalence of Trichinella spiralis in Domestic Pigs in China: A Systematic Review and Meta-Analysis
Source: Animals (Basel). 2022 Dec 15;12(24):3553. doi: 10.3390/ani12243553 (PMC9774926; doi:10.3390/ani12243553)
Supplement: Supplementary file 1 [file animals-12-03553-s001.zip › Text S2.pdf]

Quality assessment checklist.

The following items were examined and given a score based on a simple scale system (1 for "yes", 0 for "no data available" or “unclear”).

1. Was the research objective clearly stated?
2. Was the sampling area clearly described with reference to the location?
3. Was the period of the study stated?
4. Was the target sample a close representation of the general population?
5. Was some form of random selection used to select the samples？
6. Was the study with an explicit diagnostic method?
7. Was the study using analytical methods to control confounding?
8. Was the study examined several consecutive stool samples from an individual or that detected the fecal samples by multiple diagnostic methods?
