# Supplementary figures and images for: The Prevalence of Trichinella spiralis in Domestic Pigs in China: A Systematic Review and Meta-Analysis
Source: Animals (Basel). 2022 Dec 15;12(24):3553. doi: 10.3390/ani12243553 (PMC9774926; doi:10.3390/ani12243553)

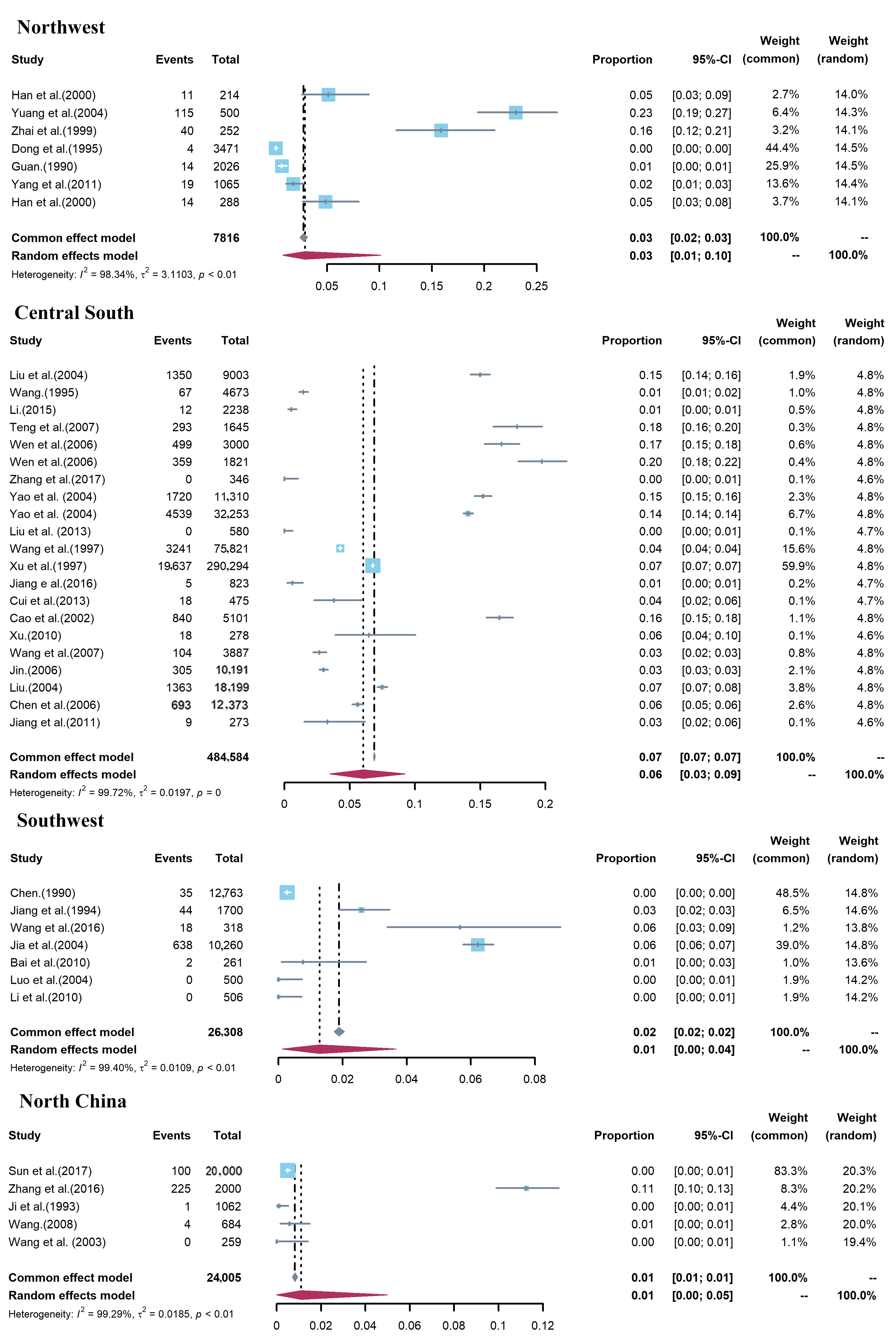

Supplement: Supplementary file 1 [file animals-12-03553-s001.zip › Figure S1.tif]

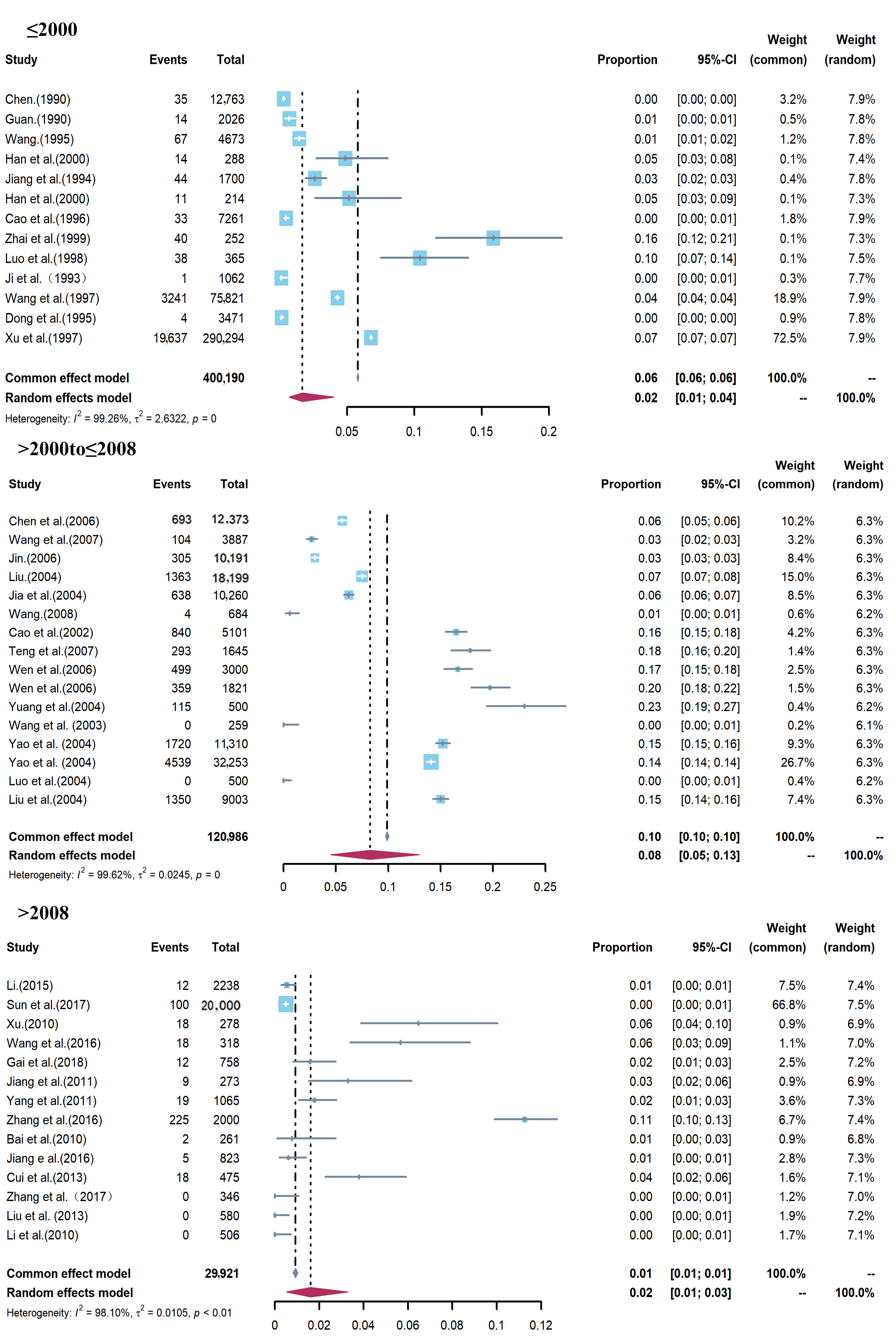

Supplement: Supplementary file 1 [file animals-12-03553-s001.zip › Figure S2.tif]

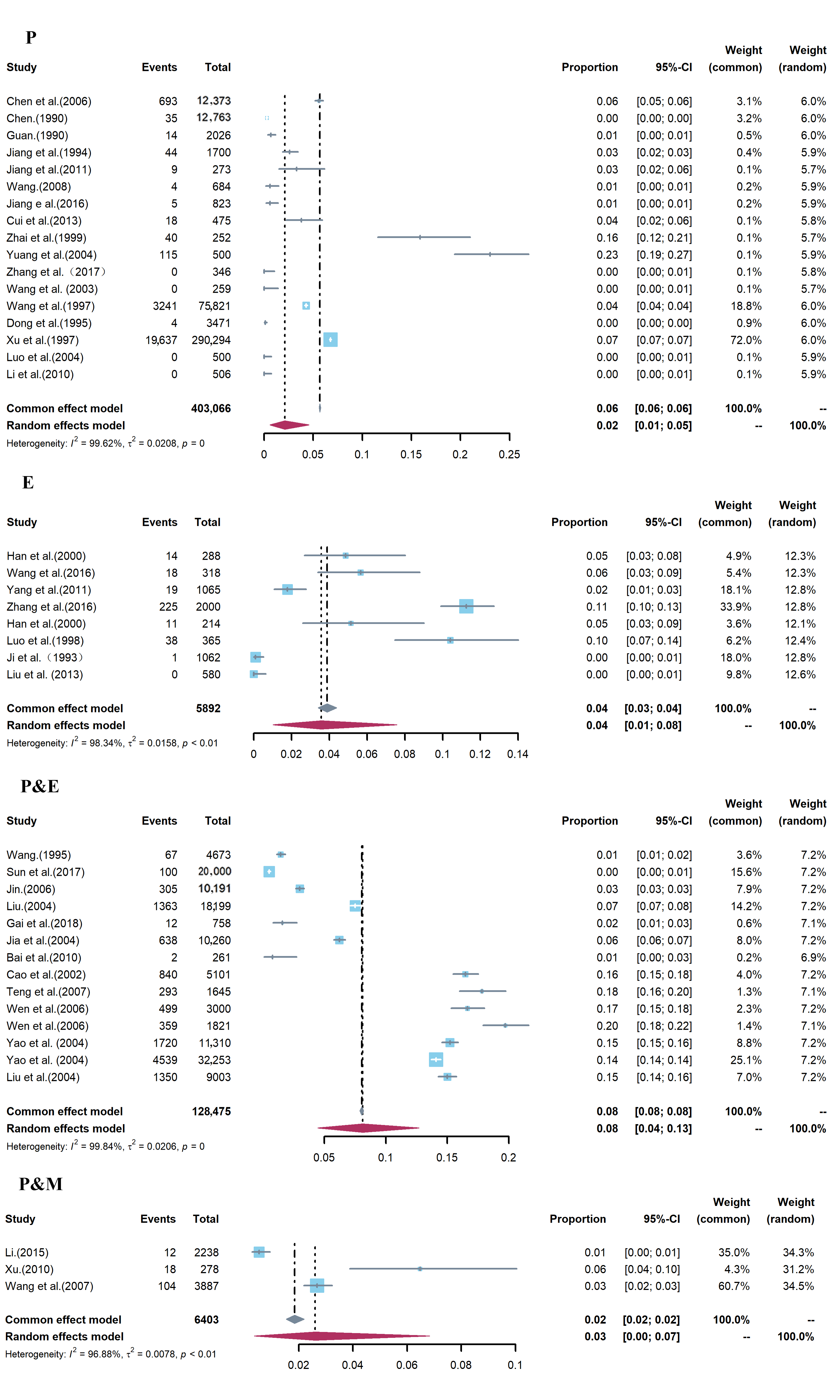

Supplement: Supplementary file 1 [file animals-12-03553-s001.zip › Figure S3.tif]

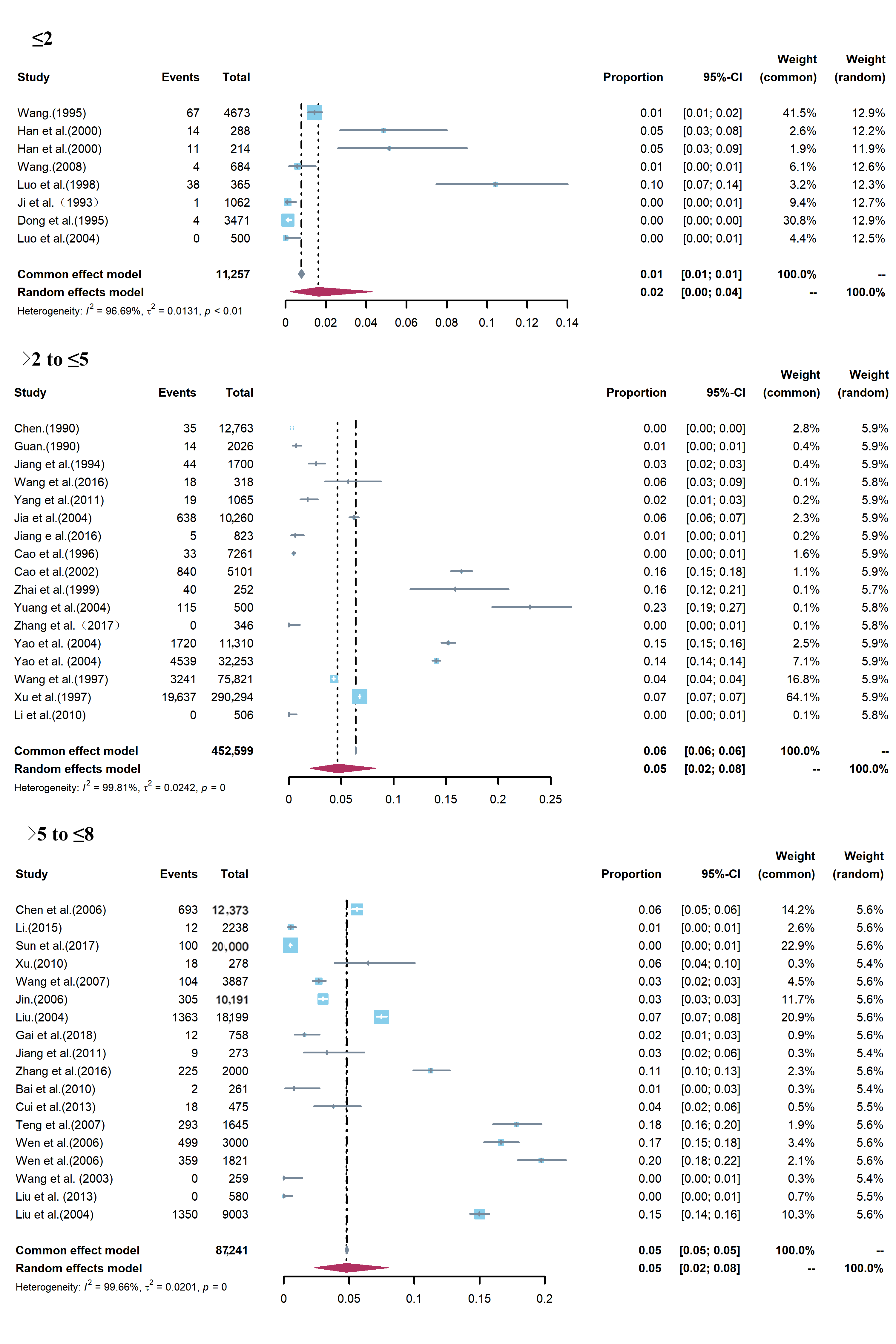

Supplement: Supplementary file 1 [file animals-12-03553-s001.zip › Figure S4.tif]

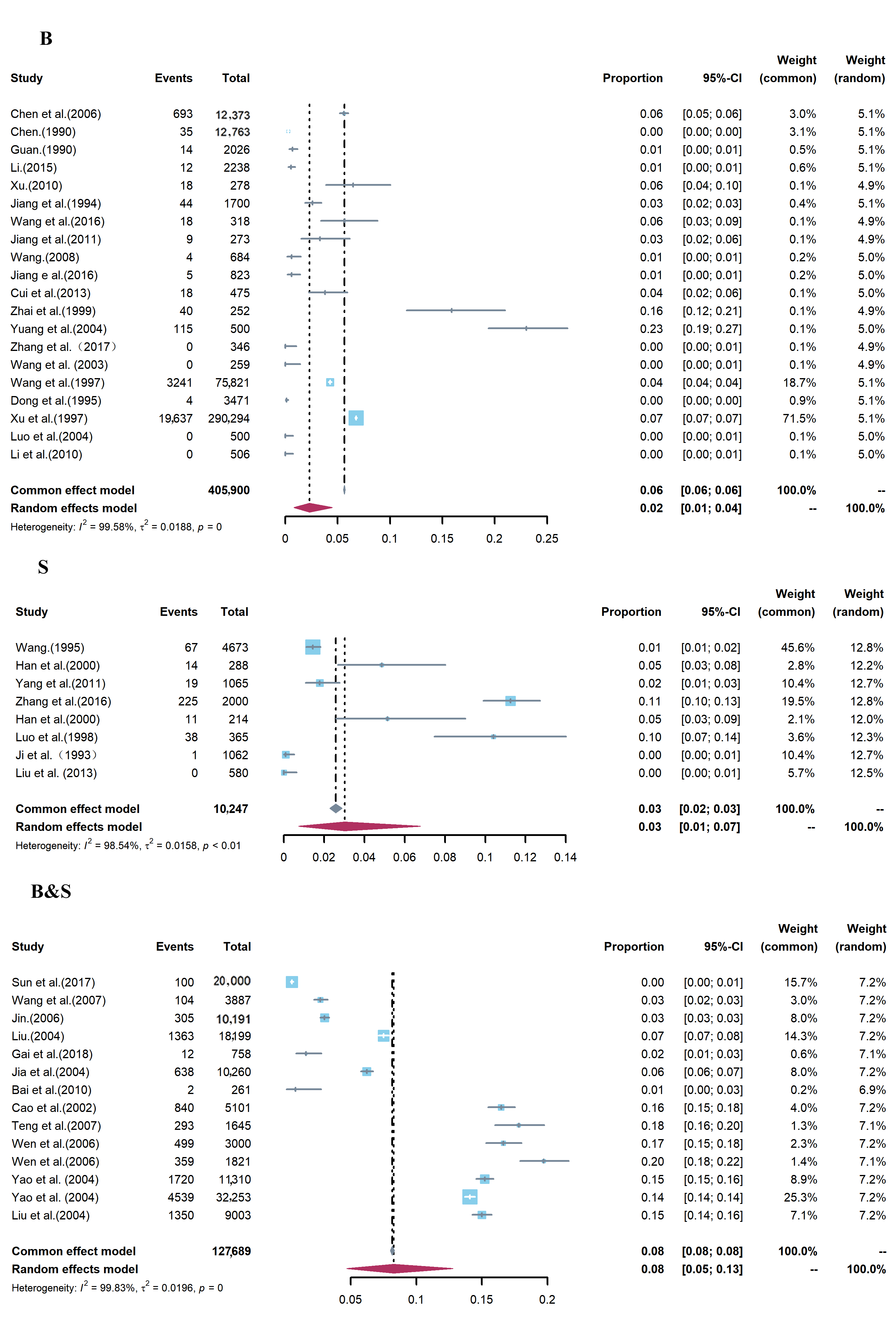

Supplement: Supplementary file 1 [file animals-12-03553-s001.zip › Figure S5.tif]

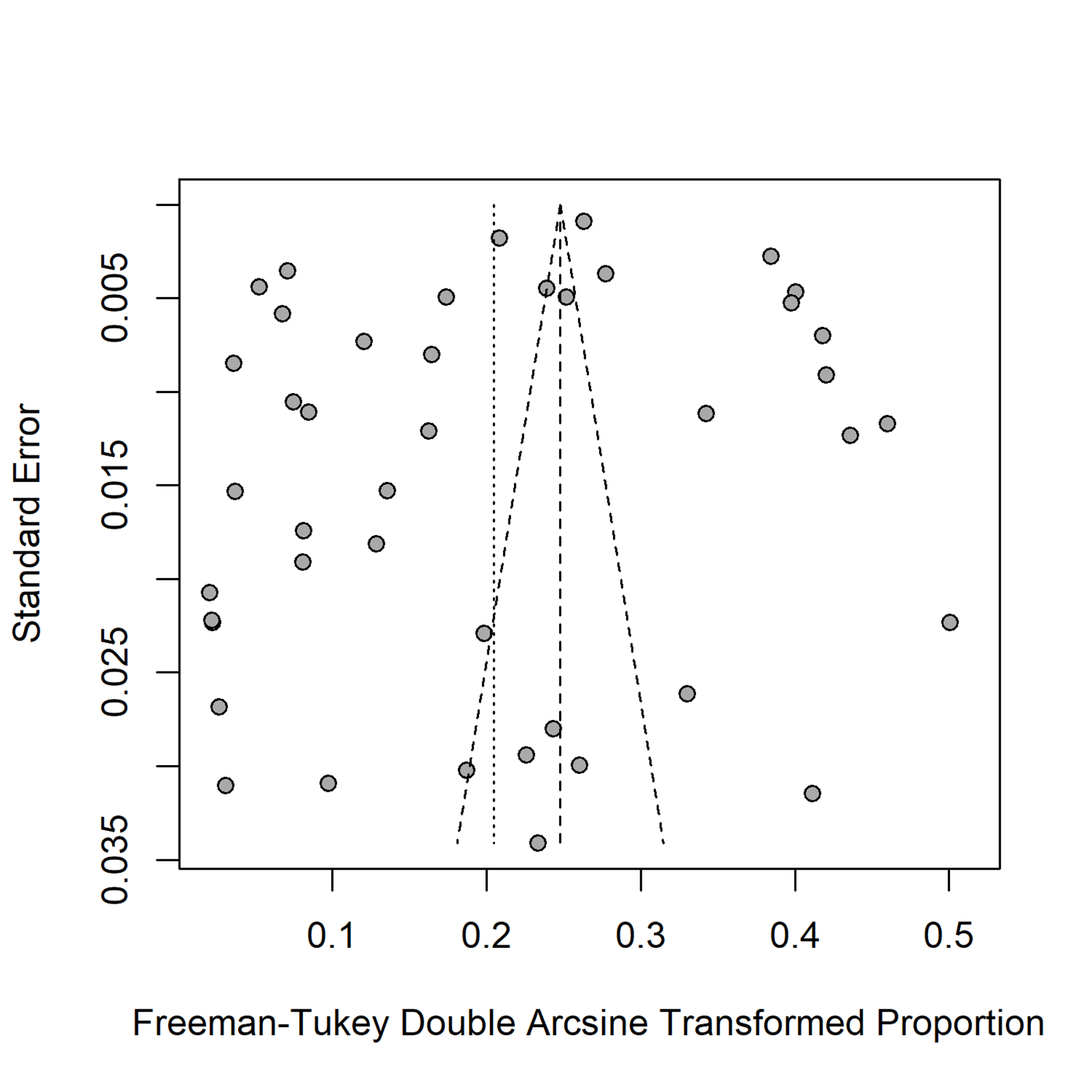

Supplement: Supplementary file 1 [file animals-12-03553-s001.zip › Figure S6.tiff]

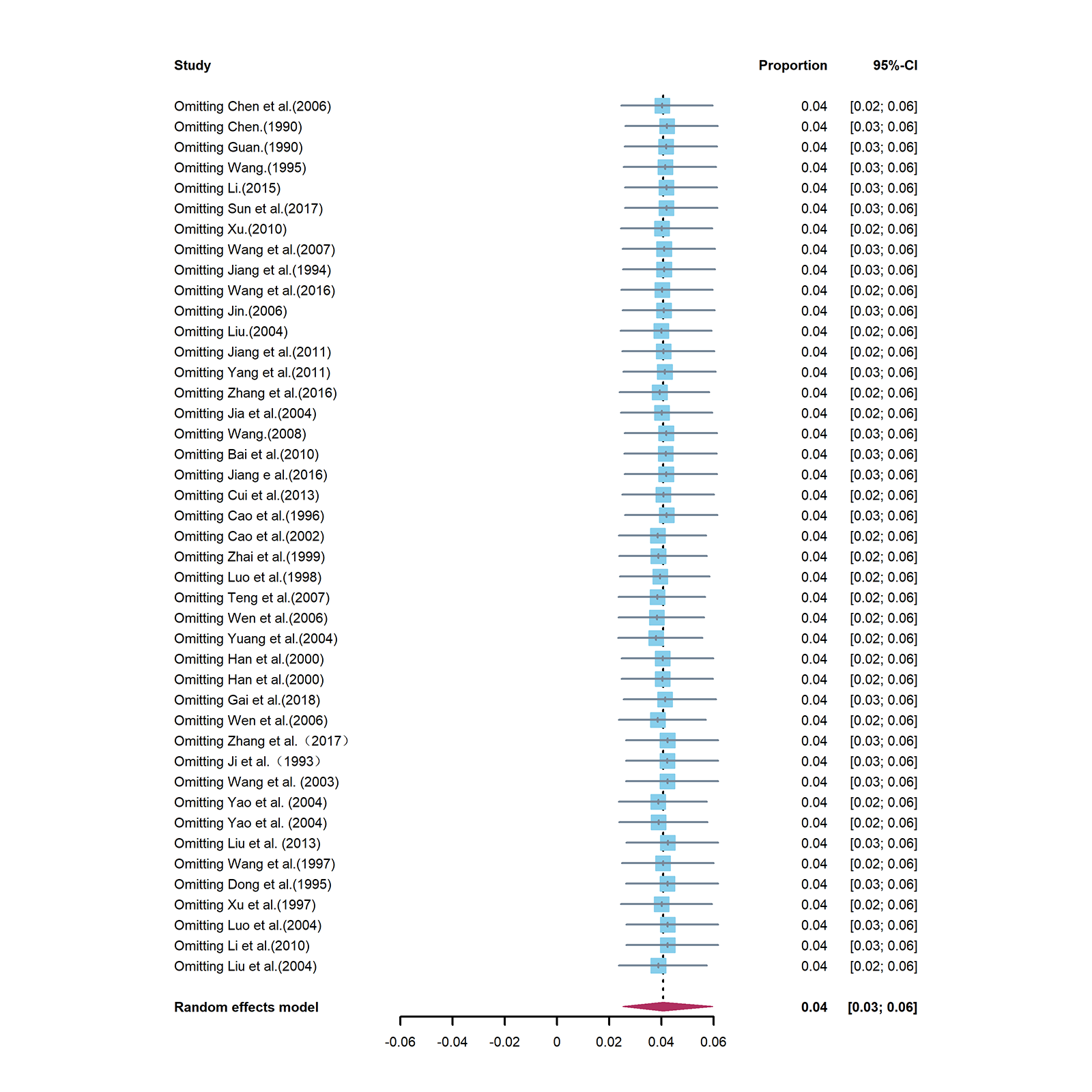

Supplement: Supplementary file 1 [file animals-12-03553-s001.zip › Figure S7.tiff]
